# Imported Hepatitis E Virus, Central African Republic, 2011

**DOI:** 10.3201/eid1902.120670

**Published:** 2013-02

**Authors:** Julie Bouscaillou, Narcisse Komas, Vianney Tricou, Emmanuel Nakouné, Benjamin Sélékon, Arnaud Fontanet, Mirdad Kazanji

**Affiliations:** Author affiliations: Institut Pasteur de Bangui, Bangui, Central African Republic (J. Bouscaillou, N. Komas, V. Tricou, E. Nakouné, B. Sélékon, M. Kazanji);; Institut Pasteur, Paris, France (J. Bouscaillou, A. Fontanet);; Conservatoire National des Arts et Métiers, Paris (A. Fontanet)

**Keywords:** hepatitis E virus, HEV, epidemiology, subtype, India, Central African Republic, migrants, viruses

**To the Editor:** Hepatitis E virus (HEV) is endemic to India (*1*,*2*) and Central African Republic (*3*,*4*), although different strains circulate in the countries. In May 2011, a case of jaundice and fever in an expatriate Indian worker (a 33-year-old man) was reported to Institut Pasteur de Bangui, Bangui, Central African Republic. HEV RNA and IgM were detected in serum samples from the patient, and liver enzyme levels were raised (alanine aminotransferase 840 U/L, reference value 11–66 U/L). Symptoms lasted for ≈10 days and resolved without specific treatment. The patient was working and living at a construction site in Central African Republic with 51 other men (22–62 years of age) from India.

 We investigated this case to determine whether it was linked to an outbreak and whether disease-control measures were needed. The protocol for surveillance and investigation was approved by the national ethical and scientific committee in Central African Republic.

Background information and blood and stool samples were obtained from the patient’s coworkers. The Bioelisa HEV IgM 3.0 kit (Biokit, Barcelona, Spain), which has sensitivity >98%, was used to test serum samples for HEV IgM; real-time reverse transcription PCR (rRT-PCR) was used to test serum and stool samples for viral RNA (*5*). Test results provided evidence of early HEV infection. Liver enzymes (aspartate aminotransferase and alanine aminotransferase) were measured in serum by using an ABX Pentra 400 benchtop analyzer (Horiba Medical, Montpellier, France). 

For genetic analysis of HEV strains from viremic study participants, we performed nested RT-PCR on serum and stool samples to amplify a 348-bp portion of the open reading frame 2 region (*6*). We directly sequenced the purified amplicons and compared the resulting sequences with HEV sequences in GenBank (*7*) and those from autochthonous HEV cases from 2008–2011. ClustalW2 (www.ebi.ac.uk/Tools/msa/clustalw2/) was used to align sequences. MEGA5 (*8*) was used to construct a phylogenetic tree (300-nt sequences) by the neighbor-joining method. The genotypes and subtypes were identified as described (*7*).

The 52 men arrived in Central African Republic in several groups during July 2010–June 2011. During May–July 2011, a total of 40 (77%) men had a febrile illness; 9 illnesses were accompanied by digestive signs or symptoms, such as nausea and vomiting (Technical Appendix Table). Only the patient whose case was reported was jaundice. Early HEV infection was biologically confirmed for 11 (21%) men, including the -patient whose case was reported; 8 of the 11 men had IgM only, 1 was HEV positive according to rRT-PCR and IgM negative, and 2 were HEV positive according to rRT-PCR and IgM positive. The 2 other men with viremia were asymptomatic, but liver enzyme levels were elevated in 1 of them. 

Illnesses in infected and noninfected men did not differ, and, except for the notified case, we cannot say with certainty that the illnesses were caused by HEV. We found IgG against HEV in 14 (34%) uninfected men, which is close to the prevalence for the general population in India (*2*).

HEV subtype 1a isolates from the notified case-patient (serum-derived isolate) and a co-worker (stool-derived isolate) were sequenced (GenBank accession nos. JN863908 and JQ074213, respectively) and found to be 100% similar and to share 97%– 99% similarity with other HEV strains in India and Nepal (Technical Appendix Figure). The sequences obtained from persons with autochthonous HEV (GenBank accession nos. JN863909, JN863910, and JQ740782) clustered with HEV subtype 1e and type 2 strains and were closely related to strains from Africa and Mexico (93% and 82% similarity, respectively). Similarity between strains was not as high for the strain from the notified case-patient and those from persons with autochthonous infection (87% similarity with the subtype 1e strain and 77% similarity with the type 2 strain).

IgM titers typically rise after the incubation period, which is >3 weeks for HEV (*9*). Thus, for the purpose of this study, we assumed that men who were positive for IgM against HEV or who had positive rRT-PCR results <3 weeks after their arrival in Central African Republic were probably infected while in India (Table, study participants 2–4 and 9). The notified case-patient and study participants 1, 5, 7, 8, and 10 had positive IgM or rRT-PCR results 4–20 weeks after arrival (Table) and, thus, could have become infected in India or Central African Republic. Study participant 6 was IgM positive >7 months after arriving in Central African Republic; thus, he was infected locally. Housing conditions for the workers were conducive to waterborne transmission of HEV, so it is likely that study participant 6 was infected with the imported strain. However, this participant was not viremic, and we cannot eliminate the possibility of autochthonous infection.

**Table:** Chronology of events and clinical test results for 11 immigrants from India participating in a study of imported hepatitis E virus, Central African Republic, 2011*

Participant, region of origin	Arrived in Central African Republic	Symptomatic period, 2011	Biological specimen collected	Test result				
IgM	rRT-PCR blood	rRT-PCR stool	AST, U/L†	ALT, U/L‡
1, Orissa	2011 Apr 29	NS	Mid Jun	+	+	+	852	791
2, Orissa	2011 Jun 4	NS	Mid Jun	−	+	−	22	14
3, Orissa	2011 Jun 4	Late Jun	Mid Jun	+	−	−	26	12
4, Orissa	2011 Jun 4	NS	Mid Jun	+	−	−	25	23
5, West Bengal	2011 Apr 5	Late Jun	Mid Jun	+	−	−	31	23
6, West Bengal	2010 Oct 10	Early Jul	Mid Jun	+	−	−	9	7
7, Orissa	2011 Apr 29	Late May	Mid Jun	+	−	−	23	18
8, Karnatanka	2011 Feb 23	Early May	Mid Jun	+	−	−	16	7
9, Orissa	2011 Jun 4	Early Jul	Mid Jun	+	−	−	19	12
10, West Bengal	2011 Apr 5	Early Jul	Mid Jun	+	−	−	24	23
NC, Orissa	2011 Apr 29	Early Jun	Early Jun	+	+	NA	2,100	840

*rRT-PCR, real-time reverse transcription PCR ; AST, aspartate aminotransferase; ALT, alanine aminotransferase; NS, not symptomatic; NC, participant whose case was notified ; NA, not available. †Reference value 5–34 U/L. ‡Reference value 11–66 U/L.

During the past few decades, concern that communicable diseases are emerging or re-emerging because of population mobility has focused mainly on mobility within industrialized countries. However, because of humanitarian and economic reasons, migration within low-income regions is also increasing. Resource-limited countries have weak infrastructures; thus, the consequences of imported outbreaks may be more serious in such countries. Our findings further demonstrate the need to improve cooperation among countries in terms of health policy, surveillance, and control, particularly in resource-limited countries. Such countries should immediately implement the International Health Regulations (2005) (*10*).

Technical Appendix Characteristics of the population studied and association with hepatitis E virus (HEV) infection, and a phylogenetic tree constructed with HEV sequences for virus strains from participant from India, persons in Central African Republic with autochthonous infection, and reference strains.
