# Supplementary material for: Imported Hepatitis E Virus, Central African Republic, 2011
Source: Emerg Infect Dis. 2013 Feb;19(2):336–7. doi: 10.3201/eid1902.120670 (PMC3559044; doi:10.3201/eid1902.120670)
Supplement: Technical Appendix — Characteristics of the population studied and association with hepatitis E virus (HEV) infection, and a phylogenetic tree constructed with HEV sequences for virus strains from participant from India, persons in Central African Republic with autochthonous infection, and reference strains. [file 12-0670-Techapp-s1.pdf]

Article DOI: http://dx.doi.org/10.3201/eid1902.120670

Imported Hepatitis E Virus, Central African Republic, 2011

Technical Appendix

Technical Appendix Table. Characteristics of immigrant workers from India who participated in a study of imported HEV infection while living in Central African Republic, 2011*

Characteristic	All, N = 52	HEV positive, n = 11	HEV negative, n = 41	p value
Mean age ± SD, years	34.8 ± 8.8	35.6 ± 12.1	34.6 ± 7.8	0.83
State (in India) of origin, no. (%)				0.83
Orissa	25 (48)	7 (64)	18 (44)	–
West Bengal	13 (25)	3 (27)	10 (24)	–
Karanataka	7 (13)	1 (9)	6 (15)	–
Bihara	5 (10)	0	5 (12)	–
Delhi	1 (2)	0	1 (2)	–
Tamil Nadu	1 (2)	0	1 (2)	–
Fever, no (%)	40 (77)	8 (73)	32 (78)	0.50
Nausea or vomiting, %	9 (17)	4 (36)	5 (12)	0.08

*HEV, hepatitis E virus.

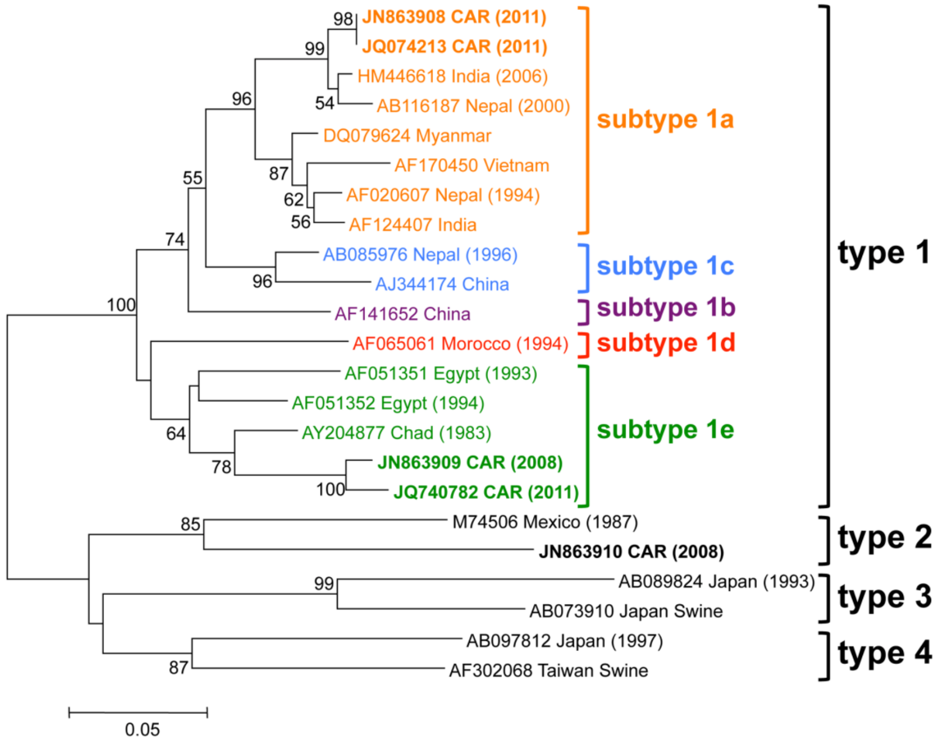

Technical Appendix Figure. Phylogenetic tree constructed with HEV sequences for virus strains from study subjects from India, persons in Central African Republic (CAR) with autochthonous infection, and reference strains. Each branch is labeled with the GenBank accession number, the country of origin, and the host (if nonhuman). Scale bar represents nucleotide substitutions per site.
